# Effects of experimental sedimentation on the phenological dynamics and leaf traits of replanted mangroves at Gazi bay, Kenya

**DOI:** 10.1002/ece3.1154

**Published:** 2014-07-22

**Authors:** Judith A Okello, Elisabeth M R Robert, Hans Beeckman, James G Kairo, Farid Dahdouh-Guebas, Nico Koedam

**Affiliations:** 1 Kenya Marine and Fisheries Research Institute (KMFRI)Mombasa, Kenya; 2 Laboratory of Plant Biology and Nature Management (APNA), Vrije Universiteit BrusselB-1050, Brussels, Belgium; 3 Laboratory of Wood Biology and Xylarium, Royal Museum for Central Africa (RMCA)B-3080, Tervuren, Belgium; 4 Laboratory of Systems Ecology and Resource Management, Université libre de BruxellesB-1050, Brussels, Belgium

**Keywords:** Mangrove trees, phenology, productivity, sediment burial

## Abstract

1. Sedimentation results in the creation of new mudflats for mangroves to colonize among other benefits. However, large sediment input in mangrove areas may be detrimental to these forests. The dynamics of phenological events of three mangrove tree species (*Avicennia marina*, *Ceriops tagal,* and *Rhizophora mucronata*) were evaluated under experimental sediment burial simulating sedimentation levels of 15, 30, and 45 cm.
2. While there was generally no shift in timing of phenological events with sedimentation, the three mangrove tree species each responded differently to the treatments.
3. Partially buried *A*. *marina* trees produced more leaves than the controls during the wet season and less during the dry season. *Ceriops tagal* on the other hand had higher leaf loss and low replacement rates in the partially buried trees during the first 6 months of the experiment but adapted with time, resulting in either equal or higher leaf emergence rates than the controls.
4. *Rhizophora mucronata* maintained leaf emergence and loss patterns as the unaffected controls but had a higher fecundity and productivity in the 15-cm sedimentation level.
5. The results suggest that under incidences of large sedimentation events (which could be witnessed as a result of climate change impacts coupled with anthropogenic disturbances), mangrove trees may capitalize on “advantages” associated with terrestrial sediment brought into the biotope, thus maintaining the pattern of phenological events.

## Introduction

The pattern of phenological events (flowering, fruiting, leaf emergence, and fall) is important in determining survival and reproductive success of plants. Moreover, leaf phenology constitutes an important aspect of studies concerning production and fluxes of organic matter in an ecosystem (Wium-Andersen and Christensen 1978; Zalamea and Gonzalez 2008). In addition, the shedding process provides a measure of organic input (Saenger and Moverly 1985; Kristensen et al. 2008; Zalamea and Gonzalez 2008). As an indicator of plant responses to environmental conditions, phenology provides insights into how plant growth can be affected by these conditions (Sekhwela and Yates 2007). Notably, changes in climatic conditions have resulted in a phenological shift in many species across various taxonomic groups due to global warming (e.g., Fitter and Fitter 2002; Visser and Both 2005; Trnka et al. 2011). Consequently, certain plant species may experience earlier or delayed bud break, flowering, and/or fruiting, hence facing an increased risk for attack by seasonal pests or unfavorable weather conditions (Rochette et al. 2004; Thampanya 2006; Henniges et al. 2007). Additionally, premature leaf fall (abscission) may also result from release of drought stress as has been witnessed in watered seedlings following severely dry conditions (Tudela and Primo-Millo 1992; Gómez-Cadenas et al. 1996).

Mangrove ecosystems form an ecotone between land and sea. As such, they are recipients of disturbances originating from both land and sea, including those due to climate change (Cahoon and Hensel 2002; Dayton et al. 2005). These disturbances (both natural and human induced) shape the ecosystem but also alter frequency and/or duration of certain processes within the ecosystem (Dale et al. 2001). An example is accretion, a natural process through which mangrove forests trap sediment facilitated by the complex aerial root network of the trees (Kathiresan 2003; Alongi 2009). Thus, mangrove forests may function as land builders by accumulating between one and eight millimeters of sediment annually (Kathiresan 2003; Smoak et al. 2013). In plantations of high density, the forests accrete up to 13 mm per year leading to an elevation increase of 2.8 mm (Kumara et al. 2010; Kimeli 2013). The deposition pattern may also vary spatially with distance from the seaward fringe (Kimeli 2013). As mangroves accumulate sediment, the long-term effect is rise in elevation which would also be important in increasing the capacity of these forests to keep pace with sea level rise (Kumara et al. 2010; Friess et al. 2012; Smoak et al. 2013).

Sediment accretion is, however, greatly influenced by disturbances including anthropogenic activities and natural disasters. For example, due to additional warming of the globe, extreme downpours have now become more frequently associated with climate change (Caldeira 2012). This may ultimately lead to an even higher sediment load brought into the mangrove areas due to flooding, with even up to 10 cm level deposit during a single downpour event (Bamroongrugsa et al. 1995). In addition, runoff from land has been aggravated by exposure of land to erosion through widespread deforestation in the catchment areas (Panayotou 1993; Pfeifer et al. 2012) as well as other human activities such as poor farming practices along the riparian zones (Harty 2009). It is therefore prudent to understand the impact of such events on important mangrove tree species' growth and development processes such as phenology.

While a number of studies on forest disturbance have focused on the ability of mangrove tree species to disperse (Rabinowitz 1978; Clarke 1993; De Ryck et al. 2012) and to tolerate temperature and moisture changes (Yáñez-Espinosa et al. 2011), relatively little has been performed to understand the impacts of siltation on growth of mangrove trees (Terrados et al. 1997; Friess et al. 2012) and none at all on its influence on the phenology of these trees. Increased sediment accretion (siltation) has been found on one hand to facilitate colonization of newly created mudflats by mangroves (Lovelock et al. 2007) and on the other hand to result in increased seedling mortality during the first few months following a siltation event (Terrados et al. 1997). Although Ellison (1998) documents other negative impacts of partial burial by sediments (die back and death) on mature trees in a number of locations, it is not clear whether there exists a threshold below which normal tree functioning is assumed before they succumb to the increase in sediment level. In this paper, crown foliage dynamics of three common mangrove species, *that is, Avicennia marina* (Forssk.) Vierh., *Ceriops tagal* (Perr.) C.B. Rob., and *Rhizophora mucronata* Lam., subjected to experimental siltation is examined over a period of 1 year. The study aims at improving the understanding of mangrove forests' resilience to extreme environmental conditions and the influence of large sedimentation events on productivity and organic matter dynamics in this ecosystem. Herewith, important information for upscaling silvicultural practices in mangrove forests, thus ensuring sustainable management of this resource, is provided. The study hypothesized that increase in sedimentation will only result in death of mangrove trees when a threshold is surpassed and that below this level the cycle of phenological events and productivity will not be affected.

## Materials and Methods

### Study site and species

**Figure 1:**
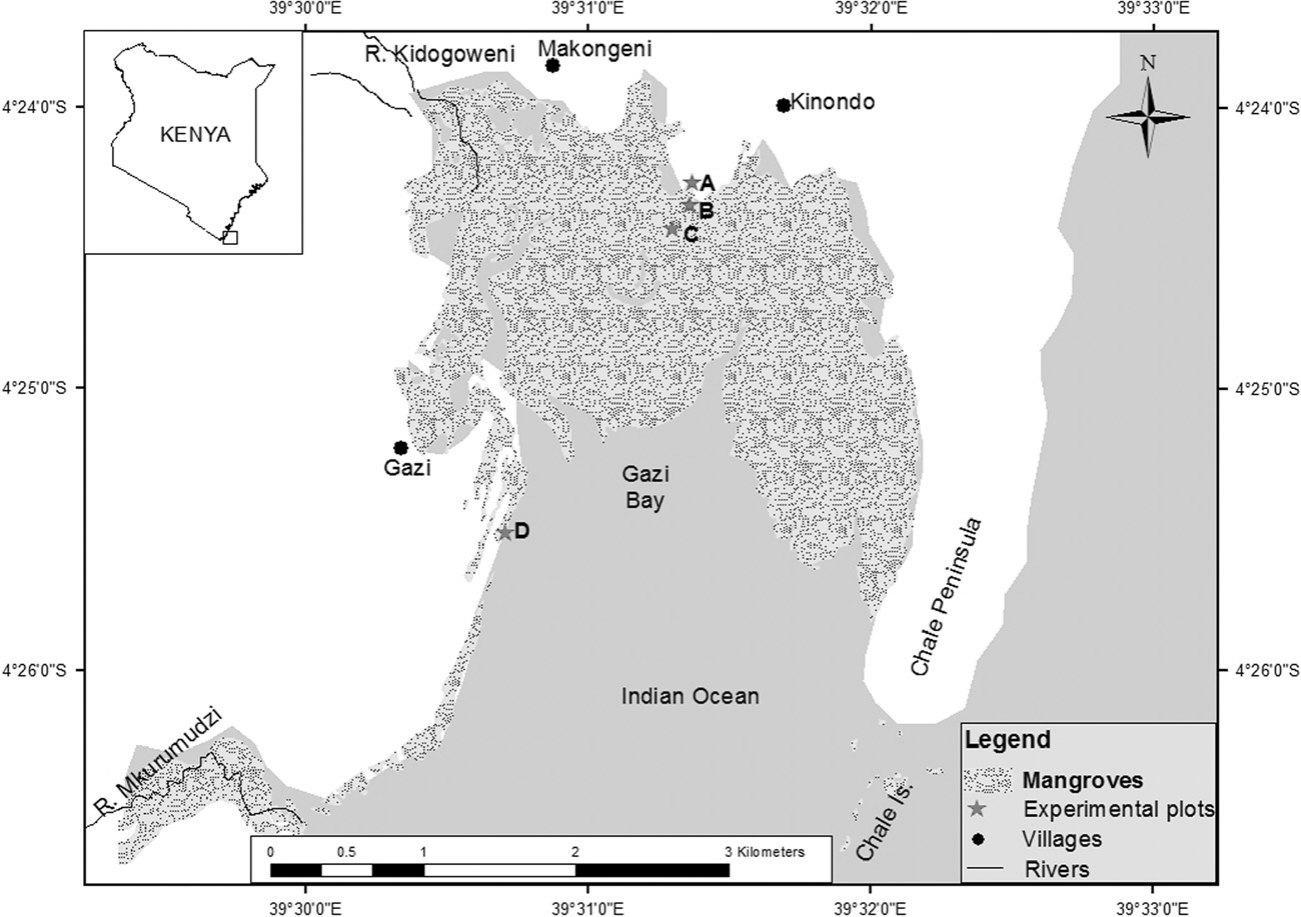
Map of Gazi Bay locating the different study sites: (A) *Avicennia*, (B) *Ceriops* landward, (C) *Ceriops* seaward, and (D) *Rhizophora*. The inset shows where the study area is located within Kenya (Kenya Marine and Fisheries Research Institute database).

The experiment was set up in plantations of *Ceriops tagal*, *Rhizophora mucronata,* and *Avicennia marina*. The trees were planted in 1994, 1998, and 2001, respectively, at Gazi bay (4°22′S, 39°30′E), 55 km south of Mombasa in Kenya (Fig. 1). Whereas the *A. marina* and *C. tagal* plantations were replanted following overexploitation that left the area bare, *R. mucronata* was replanted after the 1997–1998 El Niño rains which led to massive flooding in Kenya, resulting in mortality of mangrove trees in the study area and other mangrove areas along the coast (Kitheka et al. 2002). All the tree species were replanted in sites where they occurred before following the appropriate respective inundation classes.

**Figure 2:**
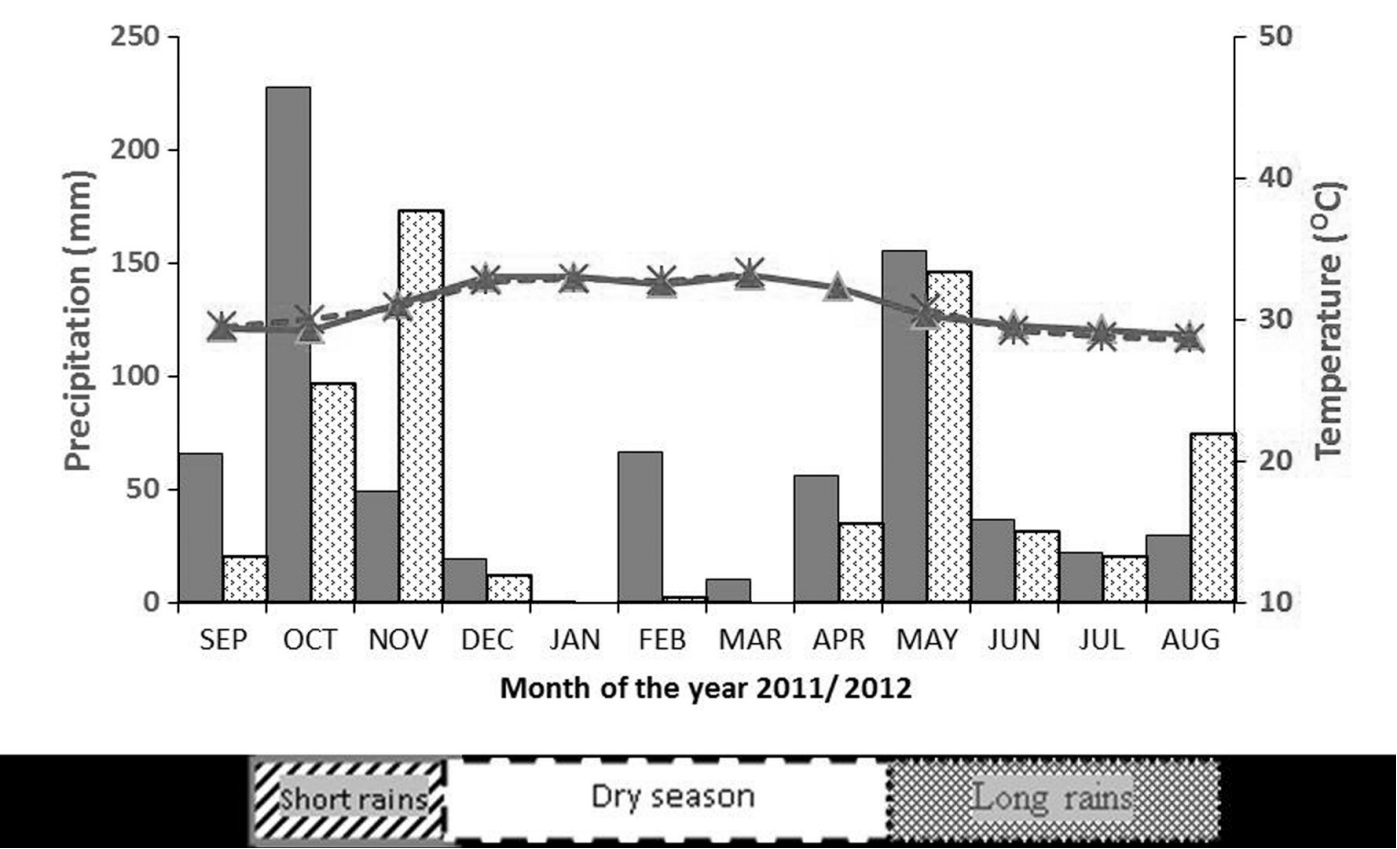
Monthly precipitation (mm – left Y axis) and mean monthly maximum temperatures (°C – right Y axis) at Mombasa from September to August 2011 [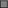/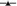] and 2012 [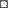/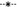] based on data from the Kenya meteorological department, Ministry of Environment and Mineral Resources, Nairobi, Kenya. The dry season (December**–**April) was relatively wet in 2011, and the transition between long and short rains (August–September) was equally wet as compared to means obtained by (Lieth et al. 1999) during the same months.

Gazi bay covers an area of 18 km^2^ (Slim et al. 1996), with a mangrove forest of approximately 615 ha (UNEP 2001; Neukermans et al. 2008). The dominant formation of mangroves in the Bay is *A. marina*, *C. tagal,* and *R. mucronata* (Matthijs et al. 1999; Neukermans et al. 2008). The climate of the area is characterized by a bimodal distribution of the precipitation (Lieth et al. 1999). A distinct dry season (December–March) is followed by a long (April–July) and a short rainy season (October–November). The study was conducted between January 2011 and November 2012 (Appendix S1) in which a similar scenario was observed (Fig. 2). However, the dry season in 2011 was relatively wet, and there was an unusually high amount of rainfall during the transition between long rains and short rains as compared to that averaged in Lieth et al. (1999). During the study period, the heaviest downpour was experienced in the short rainy season (October 2011 and November 2012; Fig. 2).

### Experimental design

**Figure 3:**
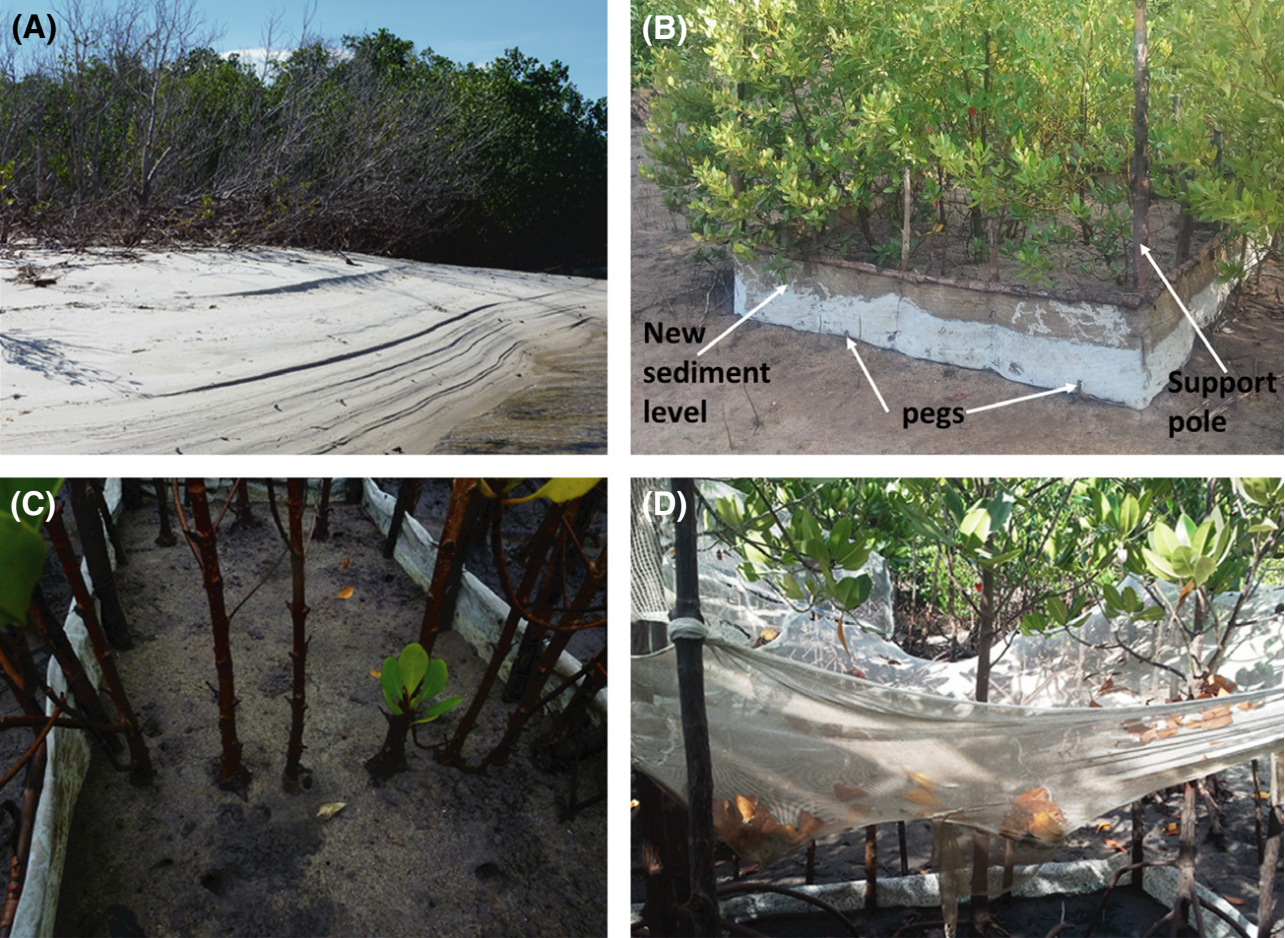
(A) A section of *Sonneratia alba* plantation in Gazi Bay dying from continuous sedimentation through wave deposition; (B) *Avicennia marina* trees partially buried with terrestrial sediment in one of the experimental plots; (C) Crab burrows in a 30-cm level of experimental sedimentation in *Ceriops tagal*; and (D)*, Rhizophora mucronata* enclosure with litter trap in one of the experimental plots. Pictures by Judith Okello 2012.

The study involved setting up experimental plots that mimic a scenario of a large sedimentation event in mangroves. Plots of 2 m by 2 m were selected within the plantations and randomly assigned to the treatments purposefully avoiding trees on the outermost edge of the plantations. The selected plots were surrounded with a netting material of 0.5 mm mesh size fixed at a depth of 10 cm around the plot and constrained to the ground using wooden pegs to avoid leakage of sediment out of the enclosure (Fig. 3). Enclosures were then filled with terrestrial sediment from adjacent upstream areas to levels of 15 cm, 30 cm, and 45 cm excluding the control, where no sediment was added, but with the same net and pegs used (Fig. 3). These sediment levels (treatments) were selected based on studies of normal accretion rates listed in a review by Kathiresan (2003), adjusted to include a few ranges within which Ellison (1998) recorded mangrove dieback and mortality in various areas. The height of each mesh was such that an allowance of approximately 10–15 cm was left at the top after adding respective quantity of sediment per treatment. During the first 2 weeks, subsidence was experienced and refilling of sediment was performed to ensure that the treatments were maintained as originally designed throughout the experiment. Benthic fauna was allowed to colonize (decapods, mollusks), and all plots were left to undergo full influence of natural mangrove processes (litter accumulation, natural regeneration, and tidal influence).

The plot sizes were designed in such a way as to include at least two central trees in the enclosure. Even when many trees were included in the enclosure, only trees that were expected to experience little edge effect as possible were selected for monitoring (Appendix S1). Eight *C. tagal* plots were created from which four were closer to the plantation edge and the other four were located about 15 m from the margin. For the purpose of this experiment, the former is referred to as landward (marked B in Fig. 1) while the latter are the seaward plots (marked C in Fig. 1). Under natural setting, *C. tagal* also occupies such positions within the tidal flat in the study site. The plantations of *A. marina* and *R. mucronata* were much smaller in areal coverage, and as such, only a single plot was laid per treatment. In all the plots, each of the selected trees was considered as replicates of each other in terms of tree responses. The selected trees were marked accordingly for subsequent monitoring. On each of these trees, six shoots were selected randomly aligned from the tree crown top to the bottom and marked at about 10 cm from the tip (reference point). The setups were performed at different times, and as such, the experiments did not run concurrently in the three study species (Appendix S1). *C. tagal* was set in December 2010, *A. marina* in April 2011, while *R. mucronata* was performed in October 2011 (Appendix S1).

### Tree variables and phenological monitoring

At the start of the experiment, tree heights were measured using a calibrated pole. Diameter measurements were then carried out at 30 cm above the highest prop root in *R. mucronata* (D_130_ sensu Brokaw and Thompson 2000). In *C. tagal* and *A. marina*, the trees were barely 2 m in height and diameter measurements were carried out at 30 cm above ground (D_30_) and at half tree height (D_1/2_). Whenever branching (multiple stems) occurred below 30 cm tree height, each of the branches (stems) was treated as a separate individual tree (Appendix S1).

On the selected tree shoots, all leaves present at the start of the experiment were numbered consecutively on the adaxial surface using a permanent marker pen. The marks were made as small as possible to avoid any adverse effect on the leaf. Monthly, new leaves were assigned the next number and nodes as well as reproductive parts located. Assessment of crown growth was performed by measuring apical increment of the same shoots as well as by monitoring development of new subshoots. Monitoring began 1 month after establishment of the experiment and lasted over a period of 1 year (Appendix S1).

### Litter fall in *R. mucronata*

Litter production of *R. mucronata* was determined by collection in 2 m by 2 m traps made of hardware net material with a 2-mm mesh size tied to poles erected at each corner of the plots (Fig. 3). These traps were suspended horizontally below the canopy of the shortest tree in the plot, but high enough to avoid soaking during high tide (Fig. 3). Weights were placed in the traps at an angle to create a depression of about 30 cm ensuring that no litter collected spilt out. Litter was emptied from the traps biweekly during the first 4 months then monthly afterward and sorted into leaves, reproductive parts (flower buds, flowers, and fruits), and woody materials (shoots, twigs, and bark pieces). The samples were weighed and then dried in the oven at 70°C until a constant mass was obtained. After obtaining the required results, all the litter was emptied back to the respective plots.

### Leaf traits

At the end of the field experiment, 20 mature leaves were selected randomly from each of the sample trees of all three species within the following conditions: (1) leaves formed during the experimental period; and (2) leaves more or less equally exposed to sunlight. The petioles were carefully cut off and each leaf scanned individually to determine the surface area using a Portable Laser Leaf Area Meter (CI-202; CID Bio-science, Washington, DC, USA). The leaves were then pooled per tree, weighed, and oven dried at 70°C until a constant mass was obtained from which the degree of succulence was determined. This was calculated by subtracting leaf dry weight per unit area from wet weight per unit area (Longstreth and Nobel 1979). The tendency to succulence provides a measure of water and nutrient preservation capacity by leaves, particularly common among trees growing in water and nutrient limited sites.

### Environmental variables

Soil physicochemical parameters (porosity, interstitial water salinity, and redox potential) were measured 3 months after establishment of the experimental setup. Repeated salinity measurements were carried out monthly for another 3 months and redox potential redone after 6 months. Interstitial water was collected using a punctured plastic tube connected to a vacuum pump, and salinity measurements were carried out with a handheld refractometer (ATAGO, Tokyo, Japan). Redox measurements were carried out using a portable pH/ORP meter with ATC and hold feature (HI 8424; Hanna instruments, Texas, USA). Sampling was carried out at 15 cm depth (top 15 cm) and at 15 cm below the interphase of the added sediment and the original ground level. Soil porosity was determined as in equation 1, following Pumpsea (2005).


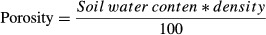
(1)

where,


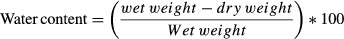
(2a)

and


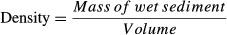
(2b)

The height above datum of the plots was determined with the help of large tracing papers painted with water soluble ink that were tied on to tree stems adjacent to each plot before high tide. The positioning of the tracing papers on the trees was estimated based on the highest tide levels obtained from tide tables (Tide table: Kilindini harbor, Kenya). Water levels after high tide were measured from the demarcation left on the tracing paper by water washing away the ink, and height above datum/mean sea level (AMSL) was calculated as follows:


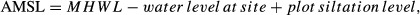
(3)

where MHWL (mean high water level) is the high tide reading from the tide table (Kilindini harbor, Kenya).

Inundation frequencies were assigned according to Watson (1928) and given in Appendix S2. Redox potential values were within the ranges associated with higher inundation classes (Matthijs et al. 1999). The redox potential ranged between −54 and 50 mV in *C. tagal* and *R. mucronata* plots and 34 and 318 mV in *A. marina* plots.

### Data analysis

Mean monthly leaf emergence, mean monthly leaf loss, leaf replacement, and shoot increment as well as percentage of shoots with reproductive parts were determined from the collected data. Leaf emergence and loss data were first tested for correlation with precipitation pattern and experimental period and categorized accordingly. In *A*. *marina,* leafing pattern was categorized based on precipitation pattern while in *C. tagal,* grouping was based on experimental time frame. Where no notable relationship was obtained (as in *R. mucronata*), data grouping was performed according to precipitation pattern. The length of time taken for a shoot to drop a leaf gained during the study period was designated leaf longevity. Fecundity was calculated as described by Hegazy (1998), where:


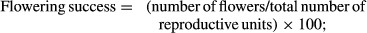



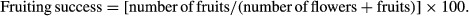


The reproductive units represent the sum of flower buds, flowers, and fruits.

Results for landward and seaward *C. tagal* were pooled for subsequent analysis in all variables except leaf flushing for which there was significant difference between the two blocks.

Data sets were tested for normality and homogeneity of variance, and as these assumptions were not met even after log transformation, Kruskal–Wallis ANOVA by ranks tests were used to determine significant differences among treatments. This was followed by a pairwise comparison of the four treatments using multiple comparison of mean ranks whenever significant difference was noted at *P* = 0.05. In addition, a Mann–Whitney *U*-test was used to test for differences in leafing between the wet months and dry months and Spearman rank-order correlations were performed to determine relationship between variables and level of sedimentation where there was significant difference across treatments. All statistical analyses were performed in Statistica 7.0 (StatSoft Inc., Tulsa, USA).

## Results

### Leaf emergence and leaf loss

**Figure 4:**
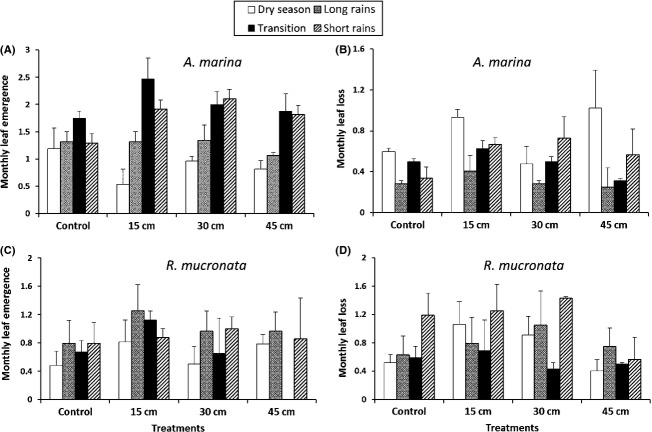
Mean (±SE) monthly leaf emergence (A,C) and leaf loss (B,D), in *Avicennia marina* and *Rhizophora mucronata* growing under different experimental sedimentation levels (15, 30, 45 cm) between June 2011 and May 2012 in *A. marina* and November 2011–October 2012 in *R. mucronata*. The wet season was taken to include the period of long rains, transition, and short rains.

A Kruskal–Wallis analysis revealed significant influence of season (H = 17.86, *P* < 0.01) as well as sedimentation levels (H = 22.98, *P* < 0.05) on leaf emergence in *A. marina*. Whereas the controls maintained similar leaf emergence rates over the different seasons (U = 135.5, *P* > 0.05 Mann–Whitney *U*-test), there were significantly higher rates of leaf emergence in the partially buried *A. marina* trees during the periods with the highest precipitation rates as opposed to the dry months (Fig. 4A; U = 302, *P* < 0.05, Mann–Whitney). There was also a general reduction in leaf emergence rates during the dry season with the controls being less affected (reduction of 17.75%) as compared to the silted trees (15 cm:– 71.88%, 30 cm:– 47.23%, and 45 cm:– 48.68%), resulting in thinner crowns. Leaf loss in this species was relatively higher during the dry season in controls and 15 and 45 cm sedimentation levels, but the same increase experienced during the short rains in the 30-cm level (Fig. 4B). Additionally, percentage number of shoots with leaf replacement rate higher than leaf loss rate was higher in the controls during the dry season and highest in the 15-cm partially buried trees during the wet months ( Fig. 6).

**Figure 5:**
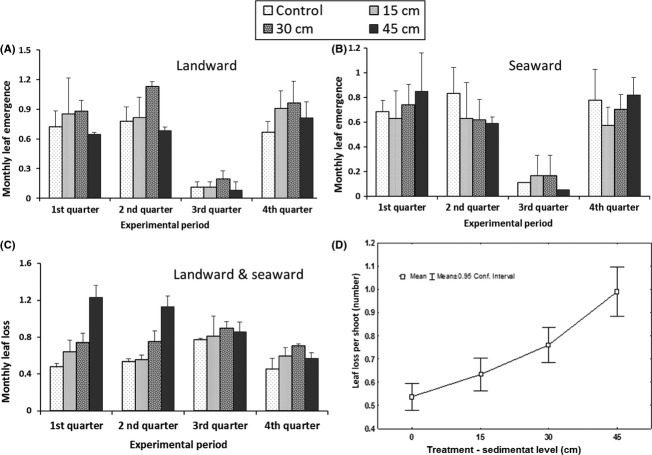
Mean (±SE) monthly leaf emergence (A,B) and mean (±SE) monthly leaf loss (C) in *Ceriops tagal* mangrove trees growing under experimental sedimentation during the first quarter (February–April), second quarter (May–July), third quarter (August–September), and fourth quarter (October–December) of the experimental period in 2011. (D) Overall mean (point) monthly leaf loss and 95% confidence interval (whiskers) among the different sedimentation treatments over the entire study period.

**Figure 6:**
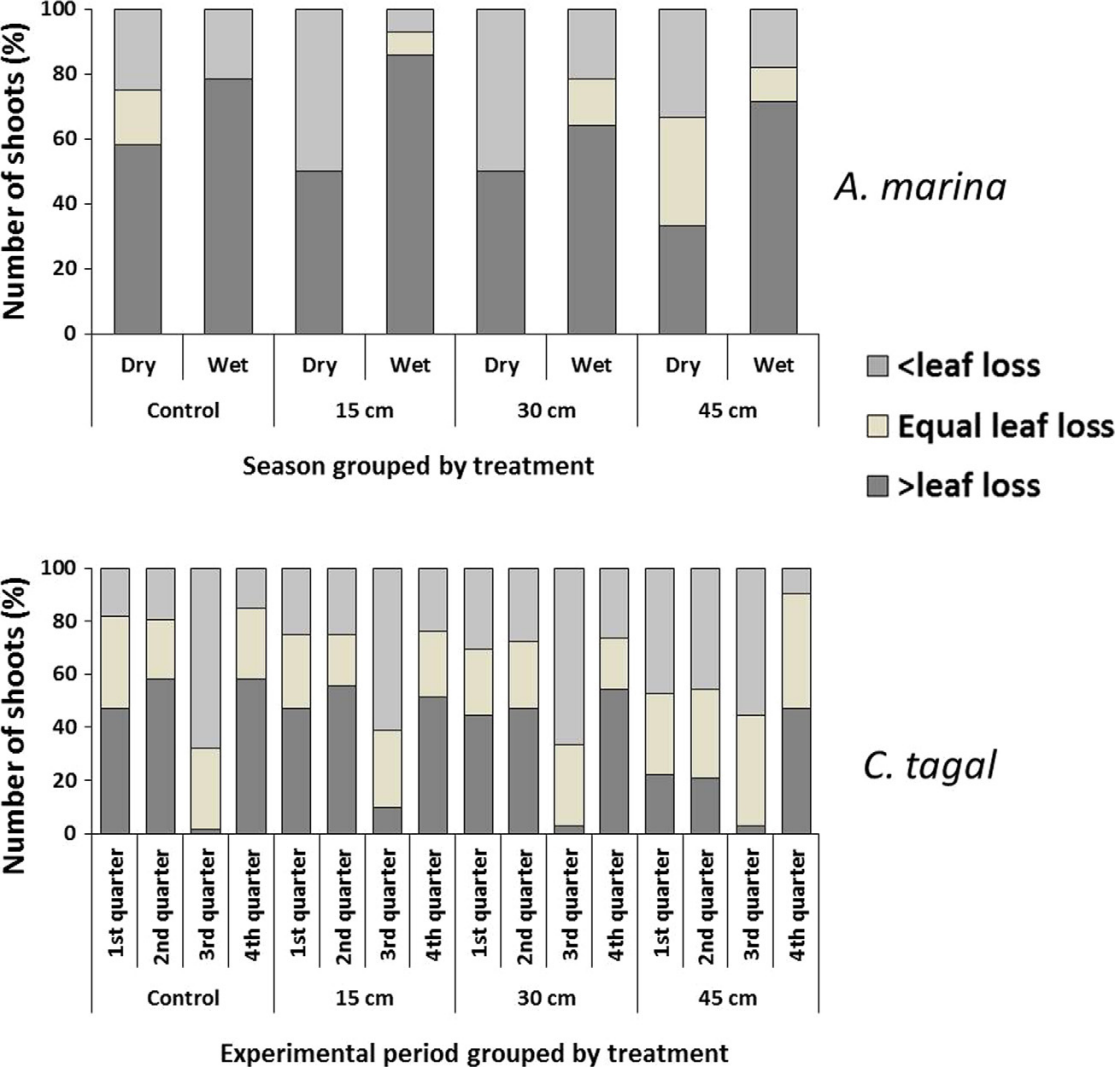
Rate of leaf replacement in *Avicennia marina* and *Ceriops tagal* expressed as a percentage number of shoots with leaf emergence rates less than, equal, or greater than the rate of leaf loss in trees exposed to experimental sediment burial simulating three levels of sedimentation (15, 30, 45 cm).

*Ceriops tagal* trees displayed a bimodal leaf emergence pattern with a significant drop in leaves gained during the third quarter of the experiment (August–September 2011) in both landward and seaward plots (Fig. 5A and B). In the landward *C. tagal*, leaf emergence was lower in the 45-cm silted trees than the rest of the treatments during the first three quarters of the experiment (February–September 2011) with a significant reduction in the second quarter (H = 15.12, *P* < 0.01). By the fourth quarter of the experiment, partially buried trees from all the levels in landward plots had relatively higher leaf emergence rates than the controls (Fig 5A). In the seaward plots, significantly lower leaf emergence rates were observed in the partially buried trees during the second quarter (H = 9.77, *P* < 0.05) with 45-cm sedimentation level showing significantly lower rates than the controls (*P* < 0.05, multiple comparison of mean ranks). Leaf loss in this species was significantly higher in the partially buried trees as compared to the controls (*P* < 0.001). This was, however, only during the early phase of the study period (first quarter; H = 31.95, *P* < 0.001; second quarter: H = 38.79, *P* < 0.001) after which all the treatments experienced similar leaf loss rates (Fig. 5C). The rate of adjustment with time was also with respect to sedimentation levels where the least buried trees (15 cm) were first to achieve reduced leaf loss by the second quarter (Fig. 5c). When seasonality was not considered, and although a Spearman rank-order correlation between levels of sedimentation and mean leaf loss per shoot was not strong (*r*_s_ (2869) = 0.1437, *P* < 0.05), the results showed a progressive increase in leaf loss with increased in sedimentation reaching exceptionally high values in the 45-cm silted trees (Fig 5D). In addition, leaf replacement rates in *C. tagal* were significantly lower in the 45-cm silted trees as compared to all the other treatments during both the first (H = 20.28, *P* < 0.001) and second quarter (H = 25.32, *P* < 0.001) (Fig. 6).

In *R. mucronata*, there was no distinct peak in leaf emergence during the entire monitoring period (Fig. 4C). In this species, there was a general alternation of peaks of decreased and increased leaf loss over the study period (H = 27.48, *P* < 0.01), with decreased leaf loss experienced in both the dry months, January–February 2011, and wet season, July–September 2011 (Fig. 4D). However, the variations were more or less uniform among the treatments with no significant difference observed in either of the seasons (*P* > 0.05).

Leaf longevity was greater than 12 months in *A. marina* and *R. mucronata,* but less than 12 months in the 45-cm silted *C. tagal* trees.

### Leaf size, succulence, and shoot elongation

Partially buried trees had leaves with larger surface area in all levels of sedimentation (*R. mucronata*) and up to 30 cm (*A. marina*, *C. tagal*) above which smaller leaves than those of controls were produced (Table 1). Leaf succulence remained similar in all the treatments as sedimentation did not show any notable influence on this parameter in the three studied species (Table 1).

**Table 1:** Mean (±SE) leaf surface area, leaf succulence, monthly shoot elongation, and fecundity in the three studied mangrove tree species after 1 year of exposure to sediment burial simulating three levels of sedimentation (15, 30, 45 cm). Marked values are significantly different from controls at *P* < 0.05 (*) and *P* < 0.01 (**)

					Fecundity	
					
Species	Treatment	Leaf surface area (cm^2^)	Leaf succulence (g dm^−2^)	Shoot elongation (cm mth^−1^)	Flowering success (%)	Fruiting success (%)
*Avicennia marina*	Control	7.46 ± 0.39	2.17 ± 0.15	1.27 ± 0.34	13.89 ± 2.78	4.04 ± 2.17
	15 cm	8.81 ± 0.41	2.07 ± 0.21	2.51 ± 0.63*	49.60 ± 12.61*	19.44 ± 10.02*
	30 cm	9.59 ± 0.51*	3.21 ± 0.84	2.35 ± 0.36	62.75 ± 12.59*	42.51 ± 21.30**
	45 cm	7.22 ± 0.31	2.26 ± 0.48	2.22 ± 0.59	66.03 ± 13.83*	27.56 ± 15.94*
*Ceriops tagal*	Control	11.61 ± 0.42	3.37 ± 0.23	0.10 ± 0.03	48.56 ± 11.23	52.4 ± 23.76
	15 cm	13.74 ± 0.63	3.60 ± 0.19	0.08 ± 0.02	53.45 ± 21.54	47.78 ± 19.83
	30 cm	12.51 ± 0.43	4.14 ± 0.34	0.08 ± 0.02	46.47 ± 14.03	51.53 ± 20.37
	45 cm	9.77 ± 0.42*	4.08 ± 0.28	0.08 ± 0.01	54.68 ± 23.89	53.45 ± 21.87
*Rhizophora mucronata*	Control	34.21 ± 1.49	3.87 ± 0.18	0.16 ± 0.02	25.09 ± 12.96	21.56 ± 23.78
	15 cm	40.44 ± 1.71	3.87 ± 0.19	0.19 ± 0.02*	65.34 ± 19.04**	54.37 ± 10.93*
	30 cm	43.25 ± 1.95*	4.04 ± 0.29	0.17 ± 0.03	26.53 ± 15.74	24.52 ± 11.67
	45 cm	39.04 ± 1.65	3.37 ± 0.04	0.18 ± 0.03	28.64 ± 12.20	22.32 ± 10.45

Shoot elongation rate was significantly higher in the 15-cm partially buried *A. marina* trees as compared to the controls (H = 14.62, *P* < 0.05), with decreased growth observed in the 45-cm sedimentation level (Table 1). Similarly, in *R. mucronata,* there were significantly higher shoot growth in the lower sedimentation level (*P* < 0.05), but the values remained similar to those in the control with higher levels of burial (Table 1). There was no distinct peak in shoot elongation observed over the experimental period. In *C. tagal,* the partially buried trees and controls had similar shoot elongation rates.

### Reproduction and litter fall

Reproduction occurred at different times of the year among the species, and there was no distinct shift in timing across the treatments in *C. tagal* (Table 2). In *A. marina*, the partially buried trees had peak flowering and fruiting earlier than those in the control while the highest silted *R. mucronata* trees fruited later into the experimental period than the controls and the 15-cm and 30-cm silted trees (Table 3).

**Table 2:** Mean (± SE) monthly litter fall in *Rhizophora mucronata* trees exposed to sediment burial simulating three levels of sedimentation (15, 30, 45 cm) over a one-year period (November 2011 to November 2012) calculated at plot and individual tree level

		Litterfall (g DW m^−2^ month^−1^)		
		
	Treatment	Leaves	Woody material	Reproductive parts
At plot level	Control	75.77 ± 11.09	0.07 ± 0.04	1.15 ± 0.39
	15 cm	79.68 ± 15.08	0.12 ± 0.08	4.67 ± 1.44
	30 cm	67.12 ± 10.84	2.79 ± 2.46	3.76 ± 1.94
	45 cm	47.76 ± 9.27	2.42 ± 2.00	2.91 ± 1.94
At tree level	Control	18.94 ± 2.77	0.02 ± 0.01	0.29 ± 0.10
	15 cm	39.84 ± 7.54*	0.06 ± 0.04	2.34 ± 0.72
	30 cm	33.56 ± 5.42	1.39 ± 1.23	1.88 ± 0.97
	45 cm	23.88 ± 4.63	1.21 ± 1.00	1.46 ± 0.97

* Marked value is significantly different from control at *P* < 0.05.

**Table 3:** Reproduction and leaf gain and leaf loss timing in the current study compared to past phenology study conducted in Gazi Bay, Kenya. The earlier studies compared phenological trends of reforested mangroves against natural forests, and *Rhizophora mucronata* reforested site is the same site where the current experiment was set up

		Peak reproduction		Leaf dynamics		
				
Species	Treatment	Flowering	Fruiting	Max leaf fall	Max leaf gain	Source
*Avicennia marina*	Natural	Feb–Mar & Dec	Apr–May	Aug–Oct	May–Aug	(Wang'ondu et al. 2010)
	Replanted	Jan–Apr & Dec	Mar–May & Jul–Sept	Feb & Aug–Nov	Feb & Aug–Nov	(Wang'ondu et al. 2010)
	Control	Feb–Apr	Apr–May	Dec–Jan	May & Aug–Oct	This study
	15 cm burial	Oct–Jan 2012	Feb–Mar	Dec–Jan	May & Aug–Oct	
	30 cm burial	Oct–Jan	Jan–Feb	Jun–Aug	May & Aug–Oct	
	45 cm burial	Oct–Jan	Feb–Mar	Dec–Feb	May & Aug–Oct	
*Ceriops tagal*	Control	Multiple peaks	Feb–Apr	Multiple peaks	Apr–Jun & Oct–Nov	This study
	15 cm burial	Multiple peaks	Feb–Apr	Multiple peaks	Apr–Jun & Oct–Nov	
	30 cm burial	Multiple peaks	Feb–Apr	Multiple peaks	Apr–Jun & Oct–Nov	
	45 cm burial	Multiple peaks	Feb–Apr	Multiple peaks	Apr–Jun & Oct–Nov	
*R. mucronata*	Natural	Jan–Feb & Dec	Mar–May	Multiple peaks	Feb & May	(Wang'ondu 2009)
	Replanted	Jan–Apr & Nov–Dec	Sept	Oct–Nov	Multiple peaks	(Wang'ondu 2009)
	Control	Feb–Apr & Aug–Oct	Mar	May & Nov	Multiple peaks	This study
	15 cm burial	Feb–Jun & Aug–Dec	Mar–Apr & Sept–Nov	Mar–May & Nov–Dec	Multiple peaks	
	30 cm burial	Feb–Apr & Aug–Oct	Mar–Apr	Mar–May & Nov–Dec	Multiple peaks	
	45 cm burial	Feb–Apr & Sept–Oct	Mar–Apr	May & Nov	Multiple peaks	

A Kruskal–Wallis analysis showed a significant effect of sedimentation level on fecundity in *A. marina* (H = 36.76, *P* < 0.001). The highest flowering success was observed in the 45-cm partially buried trees (Table 1). However, the highest fruiting success was in the 30-cm sedimentation level as more flowers were aborted in the 45-cm sedimentation level (Table 1) A post hoc multiple comparison of mean ranks revealed a significantly lower fruiting success in the controls as compared to all the sedimentation levels (*P* < 0.05). Fecundity variation in *C. tagal* had no observable trend with respect to sedimentation (Table 1) while *R. mucronata* had significantly higher flowering and fruiting success in the 15-cm sedimentation level (*P* < 0.01) than the control. However, above this level of sedimentation, reproduction did not differ significantly from the controls and each other (*P* > 0.05). There were high rates of abortion as most of the young propagules produced dropped before maturity in all the plots.

Litter production rates (g DW m^−2^ month^−1^) were highest in the 15-cm silted trees. When corrected for the number of trees per plot, the results showed a general higher leaf and reproductive litter production in the partially buried trees as compared to the controls. Production of these two forms of litter was highest with minimal sedimentation (15 cm) after which a reduction was observed, values that were still higher than the controls (Table 2). Litter production with woody material was, however, lower in the 15-cm silted plots than the other more silted (30 cm and 45 cm) ones.

## Discussion

Higher rate of leaf emergence observed in the partially buried *A. marina* and *C. tagal* in this study is in contradiction with our expectations of stress-induced mortality as a result of sedimentation as also expressed by Terrados et al. (1997) and Ellison (1998). This could be attributed to the “advantages” associated with terrestrial sediment input such as nutrient influx (Alongi et al. 2005; Lovelock et al. 2010) and greater bacterial mineralization associated with increased sediment input as suggested by Lovelock et al. (2007). This in turn resulted in the observed increased rate of leaf emergence which is also in tandem with observations made by Lovelock et al. (2006) in fertilized *R. mangle*. Nevertheless, it was striking that the “advantages” of sedimentation in *A. marina* were only expressed during the wet season. Although not systematically determined in this study, the fact that higher inundation classes are less frequently inundated and therefore trees growing here highly depend on rain water to make new biomass could partly explain the observation in *A. marina*. Additional sediment load through sedimentation raises the height above datum (Appendix S2) making the trees even more dependent on rain water thus explaining the high leaf gain during the wet season and reduced gain during dry season.

Despite the leaf emergence rates differing among treatments, the timings are comparable to those obtained in a natural landward *A. marina* site (similar inundation class as the current study) and reforested sites in a study conducted in Gazi Bay in the past (Wang'ondu et al. 2010 and shown in Table 3). *R. mucronata* leaf emergence patterns were also similar to results obtained earlier by Wang'ondu (2009) in Gazi Bay. Such multimodal peaked patterns in the congeners of the species in this study have also been observed elsewhere (Wium-Anderson 1981; Coupland et al. 2005). As such, the results in this experiment demonstrated that large sedimentation events do not affect the ever-growing nature of this species at least in the first year of partial burial.

Leaf loss is said to be part of the normal productivity cycle of an ecosystem (Zalamea and Gonzalez 2008). The observed leaf loss rates in partially buried *A. marina* and *R. mucronata* trees being comparable to those in the controls cannot therefore be pegged directly on sedimentation. However, the progressive increased leaf loss with more sedimentation in *C. tagal* (Fig. 5C) observed during the first quarter of the experiment (Fig. 5D) could be a manifestation of stress. *C. tagal* therefore seemed to be the most sensitive to sediment burial among the three study species. Additionally, *C. tagal* have been found to bear long-lived leaves (Wium-Andersen and Christensen 1978), and the contrary findings in this study are indicators of stress imposed by the increased sediment burial. There has been evidence of the negative effect of drought on leaf longevity (Mulkey et al. 1991; Mulkey et al. 1993), suggesting a possibility of physiological drought induced by large sedimentation events. However, contrary to the findings in the study by Mulkey et al. (1993) where the species with the shortest life span was the most affected, the results in the current study point toward *C. tagal* which has been found to have longer leaf longevity as opposed to *Rhizophora* and *Avicennia* (Wium-Andersen and Christensen 1978). However, the fact that all partially buried trees showed increased leaf production and leaf replacement rates similar to the controls after the second quarter (6 months into the experiment) suggests the ability to adapt to large sedimentation events. Additionally, the timing of leaf emergence was not influenced by sedimentation as all *C. tagal* trees including controls showed a bimodal leaf gain pattern (Figs 5A and B) with peaks characteristic of the species when growing in frequently inundated sites (Wium-Andersen and Christensen 1978).

Increased leaf area which was observed with minimal burial (only up to 30 cm) for all the species is in coherence with studies on mangrove growth in the New Zealand estuary (Lovelock et al. 2007). In that study, higher leaf area index was observed in sites receiving larger loads of sediment as compared to the less impacted. Possible increased hypoxia that can be caused by an increased barrier for oxygen exchange reaching the root zones as a result of sedimentation might have also been counteracted by crab burrows which were observed in the plots (Figure 3). Crab burrowing activities aid in aeration of the sediment (Smith et al. 2009) hence could have enabled the trees to grow as although increased sedimentation levels did not signify stress in any way. Additionally, crab burrowing has also been observed to help maintain relatively low levels of salinity as had been observed in a restored coastal marsh in Florida (Smith et al. 2009). Nevertheless, above the 30-cm sedimentation level, the decline in leaf area suggests attainment of a threshold while the significantly lower values in *C. tagal* from the 45-cm sedimentation level indicate an inhibitory response as a result of high burial levels.

Succulence values obtained in this study are within the range of those obtained by Wang et al. (2010) in the same species indicating no increased water or nutrient stress induced by sedimentation. Succulence is a characteristic feature of xerophytes associated with water and/or nutrient conservation as well as protection against light and herbivory (Choong et al. 1992). As mangrove trees also suffer from physiological drought (as a result of the highly saline environment), they store desalinated water. As such, they are more succulent than their neighbors, the mangrove associate (Longstreth and Nobel 1979; Wang et al. 2010) or terrestrial species, and they conserve nutrients (Feller 1996; Saenger 2002). In addition, unfavorable growth conditions may result in significant shifts in reproduction timing (Fitter and Fitter 2002) of which our results did not show such trends (*Ceriops tagal*), and where there was some degree of shift (*A. marina* and *R. mucronata*), the timings were still in concurrence with those obtained in an earlier study in the same site (Table 3). Moreover, the trees growing under increased sedimentation showed either higher fecundity (*A. marina*, *R. mucronata*) or no effect on reproduction (*C. tagal*).

## Conclusion

Large sedimentation events may not affect timing of phenological events but rather rates. This is species and site specific, but there is a general trend toward increased leaf emergence, reproduction, shoot growth, and productivity with limited sedimentation. Mangrove trees may also produce larger leaves with greater photosynthetic potential below partial burial thresholds which are species specific. Irrespective of the sedimentation level, however, if such partial burial levels are maintained at a stable level over a long period of time, the trees are able to adapt to large sedimentation events, thereby maintaining the phenological dynamics characterized with unaffected trees. In fact, it is clear from the results that while large sedimentation events may negatively affect tree development and productivity, mangroves adapt to partial burial capitalizing on advantages of accretion and thus ensure an even better growth. Sporadic large input of sediment into the mangrove ecosystems as envisaged by climate change impacts may therefore not affect mangrove growth negatively, but rather positively unless selected thresholds are exceeded above which growth and productivity declines. Future studies should, however, venture into longer monitoring periods to establish long-term effects and consider setting experiments involving gradual addition of sediment to further broaden the understanding of the impact of large sedimentation events on mangrove forests.

## Supporting Information

**Appendix S1.** Tree attributes of the three mangrove species studied in the experimental plots. **Appendix S2**. Environmental variables measured within the experimental plots.
