# Supplementary material for: Effects of experimental sedimentation on the phenological dynamics and leaf traits of replanted mangroves at Gazi bay, Kenya
Source: Ecol Evol. 2014 Jul 22;4(16):3187–200. doi: 10.1002/ece3.1154 (PMC4222206; doi:10.1002/ece3.1154)
Supplement: Supplementary file 1 [file ece30004-3187-sd1.pdf]

Effects of experimental sedimentation on the phenological dynamics and leaf traits of replanted mangroves at Gazi bay, Kenya.

Judith Auma Okello*, Elisabeth M. R. Robert, Hans Beeckman, James Gitundu Kairo Farid Dahdouh-Guebas•,and Nico Koedam•

*Correspondence author. E-mail: judith_okello2003@yahoo.com; jochieng@vub.ac.be

•Co-last author

Supplementary Information

Appendix S1. Tree attributes for the three mangrove tree species studied and the different experimental siltation levels to which they were subjected. Trees selected for monitoring were chosen in such a way as to limit as much as possible the edge effect.

Species		Treatment (sediment level)	Mean height (m)	Mean diameter (cm)		Number of trees		Monitoring period
				$D_{1/2\ ht}$	D_{30} / D_{130}	total #	# monitored	
A. marina		Control	1.2	0.8	1.4	14	4	May 2011 -April 2012
		15 cm	1.7	1.0	2.0	15	4	
		30 cm	1.4	0.9	1.6	11	4	
		45 cm	1.9	1.2	2.1	15	4	
C. tagal	Landward	Control	1.5	6.6	4.9	9	6	Jan 2011-Dec 2011
		15 cm	1.5	7.9	5.1	6	6	
		30 cm	1.4	6.7	4.7	6	6	
		45 cm	1.4	4.2	6.3	10	6	
C. tagal	Seaward	Control	1.1	6.4	4.0	8	6	
		15 cm	2.0	7.3	5.0	10	6	
		30 cm	2.1	9.2	5.9	6	6	
		45 cm	2.2	7.7	5.5	8	6	
R. mucronata		Control	3.4		11.4	4	4	Nov 2011- Oct 2012
		15 cm	3.7		15.5	2	2	
		30 cm	3.9		12.6	2	2	
		45 cm	3.4		11.8	2	2	

$D_{1/2}$ and D_{30} are tree diameters measured at half tree height and at 30 cm respectively above ground before siltation in *A. marina* and *C. tagal*, while D_{130} is diameter of *R. mucronata* measured 30 cm above the highest prop root.

Appendix S2. Environmental variables measured within the experimental plots.

Species	Treatment	Porosity		Top 15 cm Salinity (‰)		Height
		Top 15 cm sediment	Interphase 15 cm	Max	Min	above datum (m)
A. marina	Control	0.39±0.10		52	36	2.45
	15 cm	0.35±0.01	0.37±0.02	53	33	2.61
	30 cm	0.26±0.06	0.35±0.06	46	34.5	2.76
	45 cm	0.30±0.03	0.39±0.06	54	34	2.89
C. tagal Landward	Control	0.42±0.10		51	24	2.56
	15 cm	0.31±0.04	0.35±0.04	51	20	2.71
	30 cm	0.31±0.02	0.40±0.05	46	10	2.87
	45 cm	0.35±0.03	0.40±0.01	46	28	3.01
C. tagal Seaward	Control	0.38±0.04		50	27	2.48
	15 cm	0.28±0.02	0. 33±0.07	50	23	2.62
	30 cm	0.27±0.07	0.37±0.03	47	21	2.76
	45 cm	0.31±0.05	0.37±0.06	48	26	2.93
R. mucronata	Control	0.66±0.04		40	26	3.09
	15 cm	0.36±0.06	0.43±0.12	42	25	3.31
	30 cm	0.33±0.08	0.60±0.06	48	26	3.34
	45 cm	0.21±0.03	0.57±0.04	52	15	3.52

Based on height above datum, the plots are flooded 45-59 times a month (Watson, 1928).
